# Sex and age specific effects of chromosomal regions linked to body mass index in the Framingham Study

**DOI:** 10.1186/1471-2156-7-7

**Published:** 2006-01-26

**Authors:** Larry D Atwood, Nancy L Heard-Costa, Caroline S Fox, Cashell E Jaquish, L Adrienne Cupples

**Affiliations:** 1 Department of Neurology, Boston University School of Medicine, Boston, MA; 2 Framingham Heart Study, National Heart, Lung, and Blood Institute, Framingham, MA; 3 National Heart, Lung, and Blood Institute, Bethesda, MD; 4 Department of Biostatistics, Boston University School of Public Health, Boston, MA

## Abstract

**Background:**

Previously, we reported significant linkage of body mass index (BMI) to chromosomes 6 and 11 across six examinations, covering 28 years, of the Framingham Heart Study. These results were on all individuals available at each exam, thus the sample size varied from exam to exam. To remove any effect of sample size variation we have now constructed six subsets; for each exam individuals were only included if they were measured *at every exam*, i.e. for each exam, included individuals comprise the intersection of the original six exams. This strategy preferentially removed older individuals who died before reaching the sixth exam, thus the intersection datasets are smaller (n = 1114) and significantly younger than the full datasets. We performed variance components linkage analysis on these intersection datasets and on their sex-specific subsets.

**Results:**

Results from the sex-specific genome scans revealed 11 regions in which a sex-specific maximum lodscore was at least 2.0 for at least one dataset. Randomization tests indicated that all 11 regions had significant (p < 0.05) differences in sex-specific maximum lodscores for at least three datasets. The strongest sex-specific linkage was for men on chromosome 16 with maximum lodscores 2.70, 3.00, 3.42, 3.61, 2.56 and 1.93 for datasets 1–6 respectively.

Results from the full genome scans revealed that linked regions on chromosomes 6 and 11 remained significantly and consistently linked in the intersection datasets. Surprisingly, the maximum lodscore on chromosome 10 for dataset 1 increased from 0.97 in the older original dataset to 4.23 in the younger smaller intersection dataset. This difference in maximum lodscores was highly significant (p < 0.0001), implying that the effect of this chromosome may vary with age. Age effects may also exist for the linked regions on chromosomes 6 and 11.

**Conclusion:**

Sex specific effects of chromosomal regions on BMI are common in the Framingham study. Some evidence also exists for age-specific effects of chromosomal regions.

## Background

Body Mass Index (BMI) is one of the most heavily studied measures of obesity (MIM #601665). In a previous article [1] we reported substantial evidence for linkage of BMI to chromosomes 6q and 11q across six exams, covering 28 years, of the Framingham Heart Study. In that article, we found no obvious evidence for age-dependent effects of these regions, i.e. there were no monotonic changes in lodscores across the six exams. We noted that the observed variation in lodscore may be due varying sample size. In this article we present linkage results obtained from the six exams when the sample size is forced to be constant across all six exams and contrast these results with results from the full datasets. We also report sex-specific linkage analysis across the six exams.

The scientific literature on the genetics of obesity is extensive and has been reviewed in depth by Perusse et al. [2] in the latest of a series of reviews on the genetics of obesity. This review shows that there are 204 quantitative trait loci (QTL) in humans, with varying levels of support, based on published genome scans. Thirty-eight of the 204 have been replicated in 2–4 studies.

An examination of the tables presented by Perusse et al. indicate that while association studies often report sex-specific effects on obesity phenotypes, linkage results are rarely sex-specific. One of the few sex-specific linkages showed that severe obesity in women, but not men, was strongly linked to chromosome 4 in the Utah Tree Study [3]. A search of the PubMed database for sex-specific linkage of BMI yielded only one article by [4] that showed linkage of percent body fat in women to chromosome 12q and in men to chromosome 15q. The paucity of sex-specific analyses may be due to the obvious and discouragingly large degradation in power from limiting a family to one sex. In this study, our initial sample size is reasonably large (n = 1114) and we test for significant differences between sexes by computing empirical randomization tests of the difference between linkage results in men and women. Our results indicate that sex-specific effects may be common throughout the genome.

The relative effect of a gene could change over the lifetime of an individual. The Framingham study, with its longitudinal data, is one of the few studies capable of age-specific tests. In our initial analyses [1], there were no simple and obvious age-specific effects. In this study, our construction of the analysis sets allows us to begin to explore age-specific linkage. Our results indicate that age-specific effects may occur regularly.

## Results

### Descriptive statistics

Table 1 displays the descriptive statistics of the variables used here in each of the six intersection datasets. The number of individuals is constant across the intersection datasets at 1114 by design. The unusually large gap in average age between datasets 1 and 2 is due to the large time gap between the first two offspring exams in FHS. The time between the other exams is approximately four years in all cases. It is well known that BMI increases with age and is confirmed again in Table 1. The heritability of BMI increases with age and all heritability estimates are significant at p < 0.0001. The observed skewness and kurtosis are not excessive [5] therefore we did not transform the data prior to linkage analysis. The last two columns of Table 1 refer to those individuals excluded from the original datasets. For example, the original dataset 1 had 1930 individuals with phenotype information. To achieve a dataset that had observations at every exam it was necessary to set phenotypes on 816 individuals to missing. These 816 excluded individuals had an average age of 47.0 with SD = 16.9. More generally, individuals excluded from the original sets to form the intersection sets were biased toward older individuals since dropout over this 28-year span was primarily due to the death of older individuals. Mean age differences between intersection sets 1–5 and the set of individuals excluded to form intersection sets 1–5 were all highly significant (p < 0.001). As expected, mean age for intersection set 6 was not significantly different from the excluded set.

**Table 1:** Descriptive statistics for the six intersection datasets. N = 1114 in all datasets, 50% female.

Dataset	Age avg. ± SD	BMI avg. ± SD	BMI h^2^	BMI skewness	BMI Kurtosis	Original N – Excluded N	Excluded Age avg. ± SD
1 (1971–75)	35.6 ± 11.5	25.1 ± 4.0	0.45	0.7	0.6	1930 – 816	47.0 ± 16.9
2 (1979–82)	44.2 ± 12.4	25.6 ± 4.2	0.46	0.8	1.0	1764 – 650	60.1 ± 15.8
3 (1983–87)	48.6 ± 12.4	26.3 ± 4.6	0.46	0.9	1.3	1678 – 564	60.7 ± 16.5
4 (1987–90)	52.1 ± 12.5	26.9 ± 4.7	0.42	0.9	1.5	1679 – 565	60.1 ± 17.2
5 (1991–94)	55.8 ± 12.6	27.6 ± 4.9	0.50	0.9	1.3	1546 – 432	62.0 ± 17.2
6 (1994–98)	59.9 ± 12.5	28.1 ± 5.2	0.52	0.9	1.3	1401 – 287	60.1 ± 15.8

### Genome scan results

All multipoint maximum lodscores greater than 1.0 for the intersection sets are shown in Table 2. Note that several measures had multiple lodscore peaks on the same chromosome. This is reflected in Table 2 by multiple entries for the same dataset on a chromosome.

**Table 2:** All maximum lodscores greater than 1.0 for all intersection datasets

Chrom-dataset	cM	Nearest Marker	lodscore	Chrom-dataset	cM	Nearest Marker	lodscore
1-1	102.0	D1S1665	1.29	8-1	26.0	D8S1106	1.59
1–3	114.0	D1S551	1.10	8-3	39.8	D8S1145	1.78
1-1	140.0	D1S3723	1.11	8-2	41.2	D8S136	2.20
1–5	202.0	D1S518	1.15	8-4	50.4	D8S136	1.95
1–3	233.0	D1S2141	1.13	9-5	85.4	D9S1120	1.29
1–4	233.0	D1S2141	1.87	9-6	85.4	D9S1120	1.78
1–5	234.4	D1S2141	1.22	9-1	114.6	D9S938	1.42
1–6	235.8	D1S2141	1.37	10-6	35.6	D10S1430	1.81
2–3	99.0	D2S1777	1.58	10-4	38.2	D10S1430	1.71
2–6	99.0	D2S1777	1.81	10-3	51.2	D10S1423	1.52
2-2	145.0	D2S1334	1.27	10-1	63.0	D10S1208	4.23
2–5	145.0	D2S1334	1.66	11-1	106.0	D11S1986	1.60
2-1	157.2	D2S1399	1.46	11-3	119.0	D11S4464	2.43
3–5	22.0	D3S1304	1.00	11-4	119.0	D11S4464	1.71
3-3	74.2	D3S2409	1.34	11-5	119.0	D11S4464	2.29
3-2	77.4	D3S1766	1.17	11-2	124.6	D11S4464	3.51
3-2	170.6	D3S1763	1.02	11-6	124.6	D11S4464	2.70
4-3	93.0	D4S2361	2.03	14-3	70.6	D14S592	1.38
4-3	146.0	D4S1625	1.62	14-2	86.0	D14S53	1.60
4-5	146.0	D4S1625	1.25	16-2	24.4	D16S748	1.30
4-6	146.0	D4S1625	1.41	16-3	24.4	D16S748	1.20
5-3	0.0	D5S392	2.37	16-5	27.2	D16S764	1.65
5-4	0.0	D5S392	1.25	16-4	28.6	D16S764	1.62
5-5	0.8	D5S2488	1.52	16-1	41.2	D16S403	2.33
5-2	1.0	D5S2488	1.66	16-2	61.6	D16S3396	1.67
6-2	130.8	D6S1040	1.53	18-1	7.0	D18S481	1.72
6-1	149.6	GATA184A08	4.53	18-1	28.0	D18S843	1.18
6-3	166.0	D6S305	1.55	18-1	85.4	D18S1357	1.06
6-4	166.0	D6S305	1.62				
6-5	166.0	D6S305	1.15				
6-6	166.0	D6S305	3.09				

The highest lodscore over all intersection datasets was 4.53 on chromosome 6q23-25 in dataset 1. The five other datasets all had lodscores greater than 1.0 in this region. This region on chromosome 6 contains the markers *D6S1009, GATA184A08, D6S2436 *and *D6S305*. These chromosome 6 results are consistent with our results on the original datasets [1].

**Figure 1:**
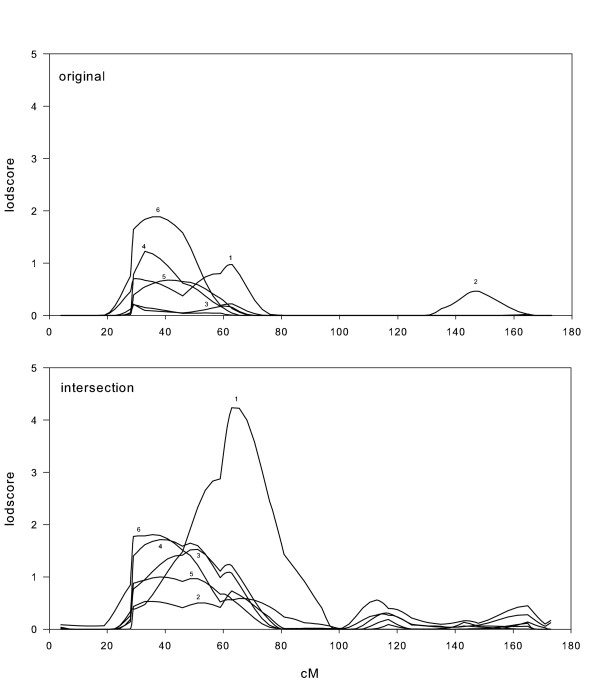
Chromosome 10 linkage results for the original datasets and the intersection datasets.

The second highest lodscore over all intersection datasets was 4.23 on chromosome 10 at 63.0 cM in dataset 1. Datasets 2–6 supported linkage in this region, with maximum lodscores of 0.73, 1.52, 1.71, 0.99, and 1.81, respectively. This region on chromosome 10 contains the markers *D10S1430, D10S1423, D10S1426*, and *D10S1208*. This is in contrast to the results on the original datasets [1]; in those datasets the maximum lodscore was 1.89 for dataset 6. For dataset 1, the maximum lodscore of 0.97 in this region of chromosome 10 from the original dataset increased to 4.23 in the intersection set. Figure 1 presents a comparison between the linkage results in the original datasets and the intersection datasets. The highest lodscore of 4.23 occurred in intersection set 1 which was 42% (816/1930) *smaller *in size than the original dataset 1 due to the exclusion of older individuals (see Table 1).

There is one other chromosomal region that shows substantial evidence for linkage across all datasets. The highest lodscore on chromosome 11q was 3.51 in dataset 2. The other five datasets (1, 3–6) supported linkage in this region with maximum lodscores of 1.60, 2.43, 1.71, 2.29, and 2.70, respectively. This region of chromosome 11 contains the markers *D11S1998, D11S4464 *and *D11S912*. These results are generally consistent with our results on the original dataset [1], however, we note that these maximum lodscores are all higher than the corresponding maximum lodscores in the original dataset, even though the datasets are all smaller.

### Age-specific effects

The randomization tests for differences between the original and intersection datasets indicated that all three chromosomes with significant linkage (6, 10, and 11) had some evidence for age-specific effects. For chromosome 6 the maximum lodscore of 4.53 at 149.6 cM was higher (p = 0.025) than expected given such a large decrease in sample size between the original set 1 and intersection set 1 (see Table 1). Results for chromosome 6 from the other five intersection sets showed no significant difference between the intersection sets and the original sets. For chromosome 10 the maximum lodscore of 4.23 at 63.0 cM was much higher (p = 0.0001) than expected given the large decrease in sample size between original set 1 and intersection set 1 (see Table 1). Intersection sets 2–5 also showed higher lodscores than expected (p < 0.05) on chromosome 10 at 63.0 cM. Intersection set 6 showed no difference. For chromosome 11 the maximum lodscore of 3.51 at 124.6 cM was higher (p = 0.032) than expected given the size of the decrease between the original set 2 and intersection set 2. Intersection sets 3–6 also showed higher lodscores than expected (p ≤ 0.05) on chromosome 11 at 124.6 cM. Intersection set 1 showed a marginally higher (p = 0.07) lodscore at 124.6 cM.

### Sex-specific effects

The approach to sex-specific genome scans taken here is simple; set all traits values for one sex to missing, and perform the genome scan in the other sex. This approach has not been generally pursued in the literature due to a large reduction in power. Since the linkage approach is based on pair-wise information, limiting the computation to same-sex pairs removes the other same-sex pairs and the discordant pairs, reducing the useful number of relative pairs by approximately a factor of four. *Under the assumption of no sex-specific effect*, we would expect such a large decrease in power that any signal that may have existed in the full intersection set would be sharply reduced or even disappear in the sex-specific subset. However, *if there is a sex-specific effect*, it will have to be quite strong to be detected in such a small subset. A sufficiently strong sex-specific linkage signal may even show higher lodscores for that sex than the full intersection datasets. It is important to bear these expectations in mind as we present the sex-specific results.

Table 3 displays all maximum lodscores greater than 2.0 from the sex-specific genome scans. A comparison of Tables 2 and 3 reveals there were 29 sex-specific maximum lodscores greater than 2.0, whereas there were only 11 maximum lodscores greater than 2.0 in the full datasets. Put another way, the genomewide evidence for suggestive linkage was much stronger in the sex-specific subsets than in the full intersection datasets, even though the sex-specific subsets were much less powerful due to smaller sample size.

**Table 3:** All maximum lodscores greater than 2.0 for the sex-specific genome scans of the intersection datasets

Chrom-dataset	cM	Nearest Marker	lodscore	Sex
1-1	86.4	D1S3728	2.01	Men
1-1	99.4	D1S1665	2.59	Men
1–2	222.8	D1S1663	2.85	Men
1–4	224.4	D1S1663	3.09	Men
1–5	224.4	D1S1663	2.33	Men
1–6	227.4	D1S1663	2.52	Men
2–4	6.8	D2S2976	2.76	Men
6-1	136.2	D6S1009	2.42	Men
7-1	109.0	D7S821	3.00	Women
8-4	19.2	D8S1130	2.67	Men
9-4	101.6	D9S910	2.33	Women
9-3	104.0	D9S910	2.67	Women
10-1	63.0	D10S1208	3.44	Women
10-3	63.0	D10S1208	2.15	Women
14-5	87.2	D14S53	2.19	Men
16-1	18.2	D16S748	2.22	Men
16-2	23.0	D16S748	2.09	Men
16-1	32.8	D16S764	2.70	Men
16-5	38.4	D16S403	2.56	Men
16-3	48.2	D16S769	3.42	Men
16-4	48.2	D16S769	3.61	Men
16-1	68.8	D16S3253	2.64	Men
16-2	68.8	D16S3253	3.00	Men
16-2	86.6	D16S2624	2.04	Men
17-1	117.0	D17S784	2.57	Men
18-1	25.0	D18S843	2.48	Men
18-3	30.6	D18S843	2.45	Men
18-4	33.2	D18S843	2.47	Men
18-2	35.8	D18S542	2.82	Men

**Figure 2:**
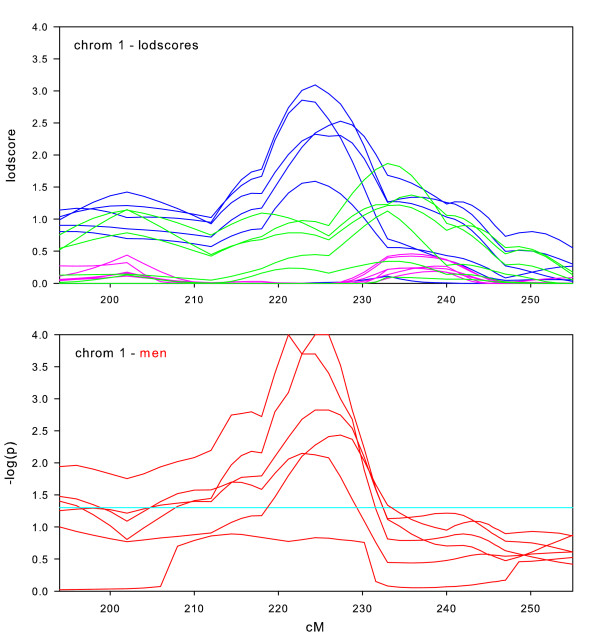
Chromosome 1 sex-specific lodscores and p-values for test of differences in sex-specific lodscores.

**Figure 3:**
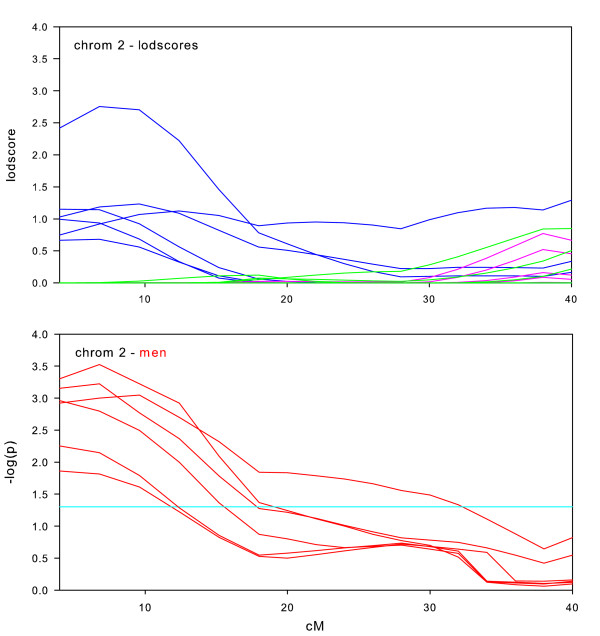
Chromosome 2 sex-specific lodscores and p-values for test of differences in sex-specific lodscores.

**Figure 4:**
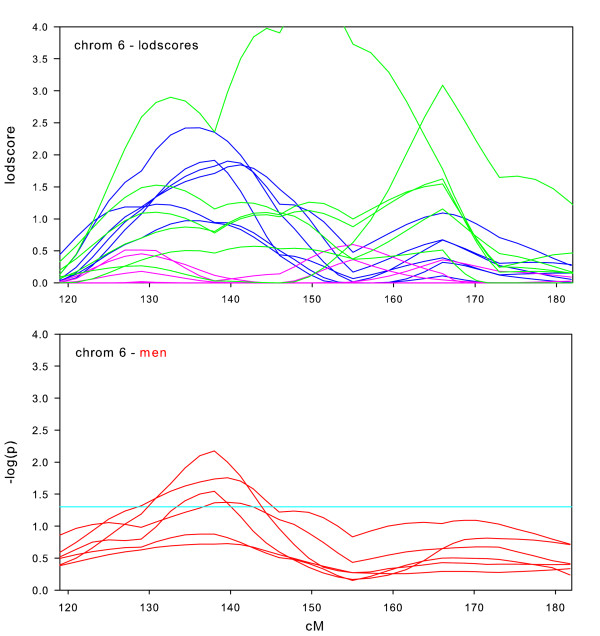
Chromosome 6 sex-specific lodscores and p-values for test of differences in sex-specific lodscores.

**Figure 5:**
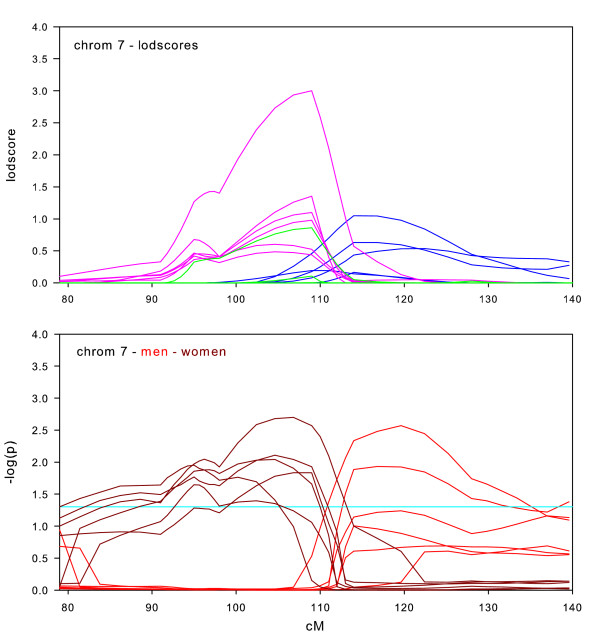
Chromosome 7 sex-specific lodscores and p-values for test of differences in sex-specific lodscores.

**Figure 6:**
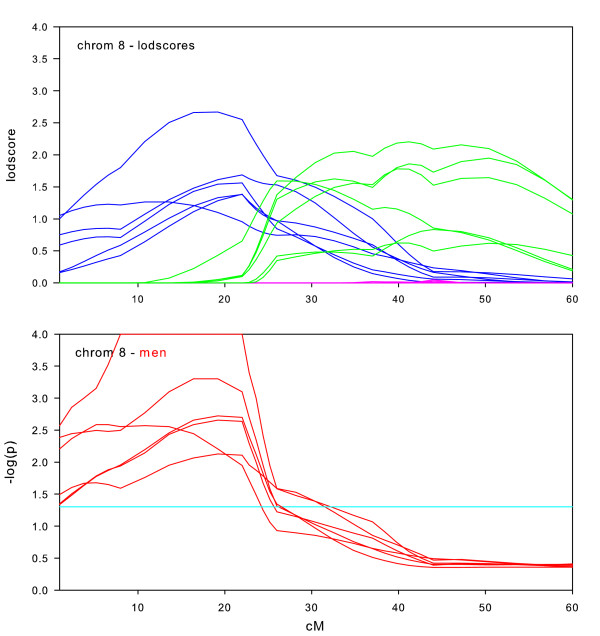
Chromosome 8 sex-specific lodscores and p-values for test of differences in sex-specific lodscores.

**Figure 7:**
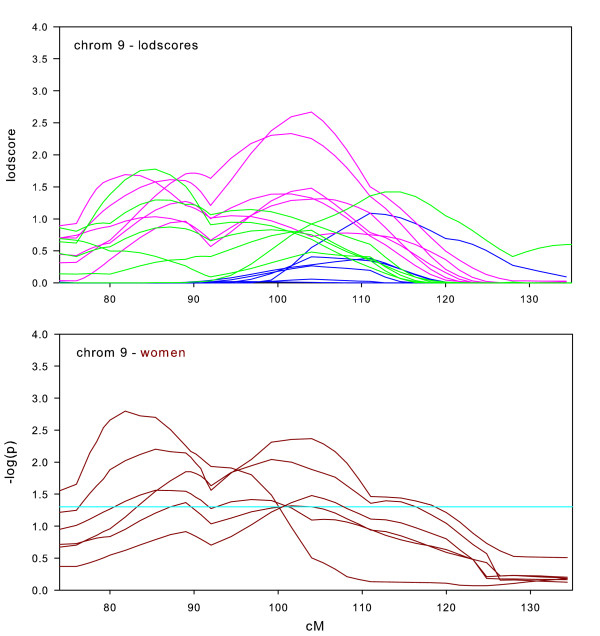
Chromosome 9 sex-specific lodscores and p-values for test of differences in sex-specific lodscores.

**Figure 8:**
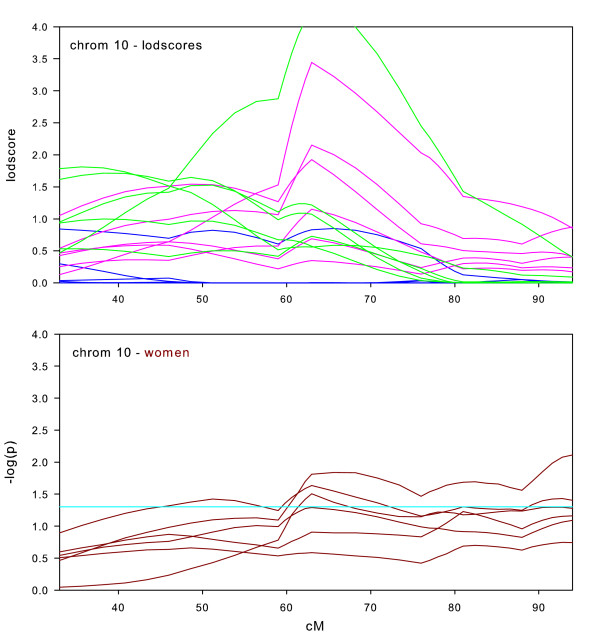
Chromosome 10 sex-specific lodscores and p-values for test of differences in sex-specific lodscores.

**Figure 9:**
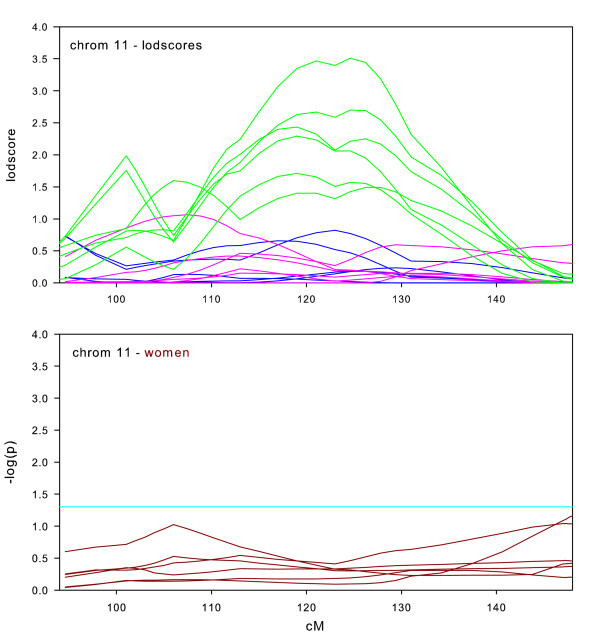
Chromosome 11 sex-specific lodscores and p-values for test of differences in sex- specific lodscores.

**Figure 10:**
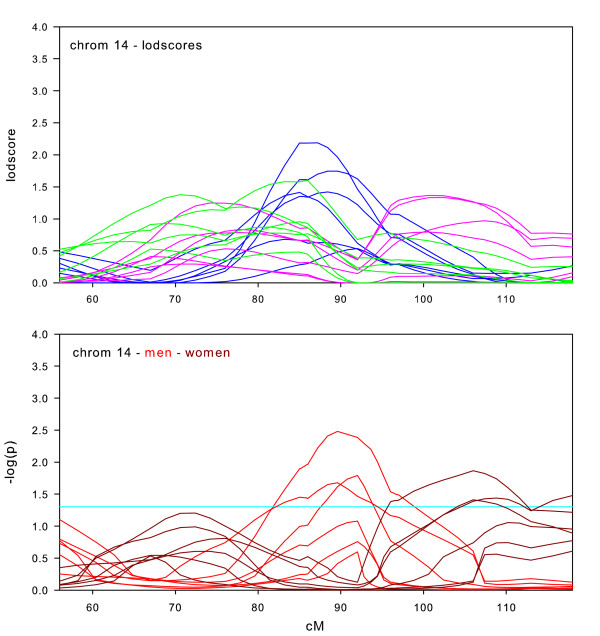
Chromosome 14 sex-specific lodscores and p-values for test of differences in sex-specific lodscores.

**Figure 11:**
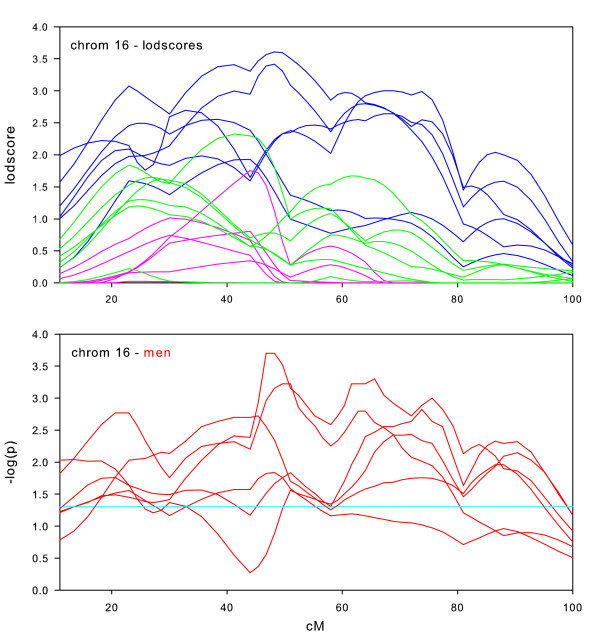
Chromosome 16 sex-specific lodscores and p-values for test of differences in sex-specific lodscores.

**Figure 12:**
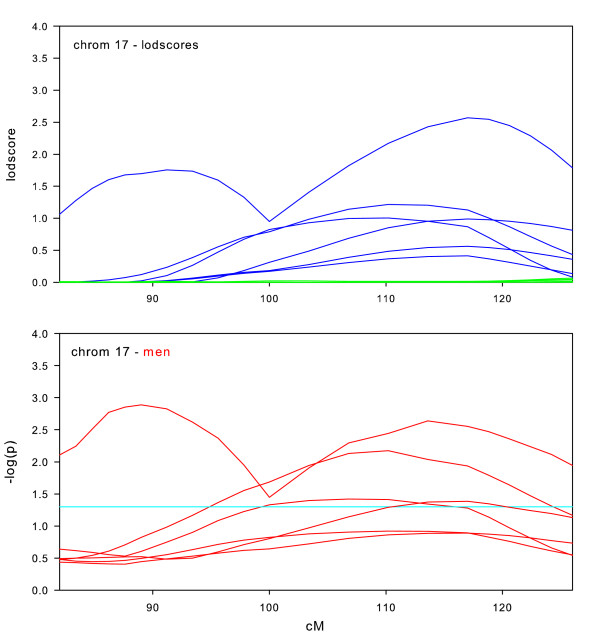
Chromosome 17 sex-specific lodscores and p-values for test of differences in sex-specific lodscores.

**Figure 13:**
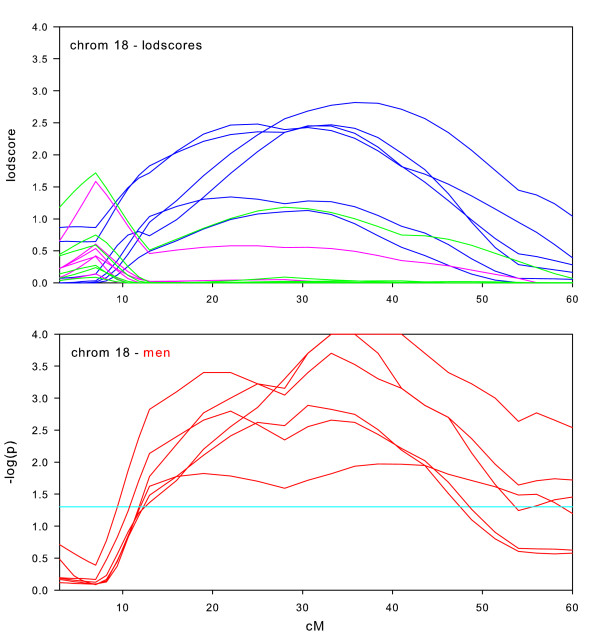
Chromosome 18 sex-specific lodscores and p-values for test of differences in sex-specific lodscores.

Randomization tests of the null hypothesis of no sex-specific effect were performed for all datasets on those chromosomes where we observed maximum lodscores greater than 2.0 in the sex-specific subsets, which were chromosomes 1, 2, 6, 7, 8, 9, 10, 14, 16, 17, and 18. We also performed randomization tests on chromosome 11, due to the significant lodscores in the full intersection dataset. For each of these 12 chromosomal regions, Figures 2, 3, 4, 5, 6, 7, 8, 9, 10, 11, 12, 13 each present a graph of the lodscore curves for all six datasets in that region and a graph of the p-values from the randomization test.

Figure 2, 3, 4, 5, 6, 7, 8, 9, 10, 11, 12, 13 are dense with information and needs careful explanation. The upper graph displays lodscore curves in the linked region of chromosome. There are 18 curves on the graph. The six curves in blue are the lodscore curves in men for each of the six subsets specific to men, the six curves in pink are the lodscore curves in women for each of the six subsets specific to women, and the six curves in green are the lodscores curves for the full intersection datasets. The peaks of the lodscore curves correspond to the values reported in Table 3. For example the highest peak on this graph in Figure 2 occurs in men at 224.4 cM with a lodscore of 3.09 (see Table 3).

The graph directly underneath the lodscore graph displays p-value curves (-log_10 _(p)) for the randomization tests of sex-specific linkage on the linked chromosomal region. Each red line corresponds to the test of the difference in lodscores between men and women (shown in the graph directly above) for one of the datasets. For example, a value of 3 on the graph corresponds to a p-value of 0.001. The light blue horizontal line represents a nominal p-value of 0.05.

Sex-specific results for chromosome 1 are shown in Figure 2. Men (blue curves) have consistently higher maximum lodscores than the full datasets (green curves) and the full datasets have consistently higher maximum lodscores than women (pink curves). The chromosome 1 results are the expected pattern for a strong sex-specific effect. The location of the maximum lodscores in men is between 220 cM and 230 cM. However, the maximum lodscores in the full dataset is between 230 cM and 240 cM. This discrepancy in location, if confirmed, has important implications for fine mapping and positional cloning. The p-value graph shows the observed differences between men and women are significant (p < 0.05) for five of the six datasets with the stronger linkage signal in men.

Sex specific results for chromosome 2 are shown in Figure 3. All 18 lodscore curves are present; it only seems less since several of them had all zero lodscores. Only one dataset in men (blue curve) had a maximum lodscore greater than 2.0. Nonetheless, the sex-specific tests showed a significant larger effect in men for all datasets, with p-values less than 0.001 (-log(p) > 3.0) in three of the six datasets.

Sex-specific results for chromosome 6 are shown in Figure 4. The maximum lodscore in the full datasets was 4.53 at 150 cM (off the top of this graph). The sex-specific results around 150 cM were both much lower and the differences were not significant, indicating that the strong linkage in this area is not sex-specific. However, there is a significant sex-specific difference around 140 cM for four of the six datasets, with the maximum lodscore in men of 2.42 at 136 cM (see Table 3). These results could indicate a second sex-specific locus around 140 cM.

Sex-specific results for chromosome 7 are shown in Figure 5. Linkage of BMI to chromosome 7 has been replicated in the literature [6-8], however, the location of the linked regions have all been slightly different. In Framingham, the full datasets show essentially no linkage. The sex-specific results however are suggestive of two linkage regions, one in women (maximum lodscore 3.0 at 109 cM) and a weaker one in men around 114 cM. The tests for sex-specific differences were significant for both men and women. To make this clear, we plot p-value curves for both men (red curves) and women (dark red curves). The possibility of two loci, one in men and one in women, may clarify some of the conflicting linkage results of BMI to chromosome 7. It is also interesting to note that the three datasets that show some linkage in men are the three oldest datasets.

The sex-specific results for chromosome 8 (Figure 6) indicate significant differences between men and women at 15–25 cM with the stronger signal in men. All six datasets were significant in this area; two datasets were highly significant (p < 0.001).

A region on chromosome 9 (Figure 7) was one of the few that showed consistently stronger linkage in women than in men. From the graph, it is clear that almost the entire linkage signal in the full datasets originated in women. All six datasets showed significantly stronger linkage in women than in men at some point in this wide region (75–120 cM).

As already discussed, there was a maximum lodscores of 4.23 on chromosome 10 (Figure 1). The sex-specific graphs (Figure 8) make it clear that most of that strong linkage signal originated in women as the women-only subset of that dataset had a maximum lodscores of 3.44 at the same location. Only three of the six datasets showed significant differences in lodscores between men and women. This may indicate that men do contribute to the overall linkage, albeit weakly.

We include chromosome 11 (Figure 9) in the sex-specific linkage only due to the high lodscores in the full datasets. It is obvious from the graph in figure 9 that this linkage is not sex-specific. This is confirmed by the tests of the difference between men and women in which none of the datasets showed any significant differences. It is interesting to note that this chromosome conforms to prior expectation when there is no sex-specific linkage, i.e. the large reduction in power due to the smaller sample size caused the linkage signal to disappear in the sex-specific subsets.

Sex-specific results on chromosome 14 (Figure 10) reveal two areas of significant differences in sex-specific lodscores. In men, there is some evidence for linkage at 85–95 cM. In women, there is slightly weaker evidence at 95–110 cM. The p-value graph indicates three datasets were significant in men and three in women. The three datasets where linkage is significantly greater in women than men are the three youngest datasets, whereas the three datasets that show the linkage significantly greater in men are the three oldest datasets. This is tentative evidence of an interaction between sex and age.

Sex-specific results on chromosome 16 (Figure 11) provide the strongest evidence for sex-specific linkage. The subset limited to men had maximum lodscores of at least 2.0 in all six datasets and at least 3.0 in three of the datasets. The linkage was significantly stronger in men across an 80 cM region (10 cM-90 cM) of chromosome 16. The size of this region and the multiple peaks suggests multiple loci affecting BMI.

Sex-specific results for chromosome 17 (Figure 12) are most remarkable for showing no linkage at all in the full datasets. Four of the sex sex-specific subsets show that linkage in men is significantly stronger than in women. In fact, women show no evidence for linkage at all. This result indicates that linkage in one sex can be completely masked by the absence of linkage in the other sex.

Sex-specific results for chromosome 18 (Figure 13) are similar to chromosome 17 in that the full datasets show no real evidence for linkage, but the sex-specific subsets provide evidence for linkage. In this case, four of the six sex-specific subsets have maximum lodscores greater than 2.0 in men, and practically no signal at all in women. This difference between men and women is significant in all six datasets across a large region of the chromosome.

## Discussion

We found strong evidence (maximum lodscore 4.23) for linkage of BMI to *D10S1208*, which is at 63.0 cM in the youngest dataset. The next highest peak, telomeric to this peak, was in the oldest dataset. This result replicates linkage already found in the same region for four independent cohorts. A study of French families [9] found significant linkage of BMI to *D10S197 *at 52 cM. A study of young German families [10] replicated this result by finding evidence for linkage at D10S1781 at 61 cM. Finally, a joint study of both European-American families and African-American families [11] also confirmed linkage in both ethnic groups at *D10S197 *with a secondary peak at *D10S208 *(61 cM). We believe that this range of peak lodscores from 52 cM to 63 cM is within the bounds of error for localization of a QTL across multiple independent studies.

The evidence for widespread sex-specific linkage effects on BMI of varying chromosomal regions in the Framingham Heart Study seems overwhelming. Regions on 11 different chromosomes showed significant differences between maximum lodscores in men and women. In nine of those regions the sex-specific maximum lodscore was higher than the maximum lodscore in the full dataset, in spite of the fact that the full datasets had larger sample sizes. In these cases it seems clear that the presence of the unlinked sex in the full dataset was actually attenuating the linkage signal and lowering the lodscore. The ubiquitous nature of the sex-specific results has implications for other family studies of BMI, especially those with sufficient sample size to have viable sex-specific subsets. We believe that linkage analysis of sex-specific subsets should become standard practice for BMI.

It is puzzling that, of the eleven sex-specific regions, eight were linked to men and only three to women. The two significant lodscores in women (see Table 3) both occurred in the youngest dataset, when few of these women had gone through menopause. The loss of that linkage signal over time may be due to the onset of menopause in this cohort. However, it may also be due to the generally increasing genetic heterogeneity with time and the sex-specific effect may be unrelated to menopause. Perhaps men are just simpler than women.

The evidence for age-specific linkage effects on BMI of chromosomal regions in the Framingham Heart Study, while not as ubiquitous as the sex effects, is still strong. It should be emphasized that, for each of the three chromosomes (6, 10, and 11) there were six randomization tests. Each test was a comparison between the original dataset and the intersection dataset. The strongest evidence for differences between the original and intersection datasets tended to occur in datasets 1–3, which had the largest age differences between original and intersection. This was especially true for chromosome 10 where a maximum lodscore of 0.97 in the older original dataset 1 actually increased to 4.23 in the younger intersection dataset 1. As Table 1 shows, the average age difference between those individuals included in intersection dataset 1 and those individuals excluded was 11.4 years. Conversely, the weakest evidence for differences between the original and intersection datasets tended to occur in datasets 4–6, which had the smallest age differences between original and intersection datasets. Indeed, dataset 6, which had the smallest age difference between original and intersection datasets, did not show any significant difference on any chromosome.

The tendency for stronger linkage in younger individuals extends to the sex-specific subsets. Table 3 shows that 10 of the 29 maximum sex-specific lodscores greater than 2.0 occurred in the first (youngest) dataset, whereas only 1 occurred in the sixth (oldest) dataset.

Table 1 indicates that heritability of BMI increases with age. It therefore might seem paradoxical that evidence for linkage is stronger in younger datasets where there is lower heritability. We conjecture that genetic heterogeneity increases with age, i.e. more genes affect BMI in an older sample than in a younger sample. Unfortunately, linkage methodology is not robust with respect to increasing genetic heterogeneity. Thus, even though the overall genetic affect is larger in older samples, the *detectable single locus effects are more likely to be in younger samples *simply because fewer genes affect BMI at younger ages. If this conjecture is correct, then it is unfortunate that most studies of common disease are designed to maximize heritability and therefore have ascertained older subjects.

The statistical test we use to infer significant sex-specific and age-specific is a randomization test, which is the emerging standard in complex genetic analysis. However, the difference in the way we used the test here should be noted. The sex-specific test structured the randomization directly, based on sex. The age-specific test was indirect; the randomization was structured to achieve a uniform sample across all six datasets. The inferred age-specific effect was secondary to the randomization and based on observed age differences between the original sets and the intersection subsets. Thus, while we believe the age difference is the most parsimonious explanation, it is certainly possible that some other unobserved factor is responsible for the significant difference in the two datasets. Thus, the direct evidence for the sex-specific effects should be regarded as stronger than the indirect evidence for age-specific effects.

This study offers insight into the replication problem. The inability to replicate significant linkage results has bedevilled this field for years and has been a serious hindrance to progress. Some of the reasons cited for lack of replication have been different study designs, different ascertainment schemes, ethnic differences, genetic heterogeneity, and random error. This study of BMI adds two factors to that list. The first factor is sex. A study that is exclusively or predominantly of one sex may show quite different patterns of genetic effect than a study that is of the other sex or both sexes. The second factor is age. If it is generally true that some single locus effects are more easily detectable in a certain age range, then age differences between studies could hinder replication.

The unique nature of the Framingham cohorts needs to be emphasized. Unlike many family studies Framingham is population-based and not ascertained on a particular disease trait for which obesity may be a risk factor. The intersection datasets, which are crucial to this study, required, by definition, that each individual be present at all six examinations. The natural consequence of this design is that the subjects of this study are the youngest and healthiest individuals in what was already a study of normal health. A final caveat about this study is that the first dataset, where so much of the significant linkage was detected, is from examinations that took place in 1971–1975. It is unlikely that any current study could replicate the dietary and physical activity patterns that were prevalent at that time.

## Conclusion

Sex and age specific effects of chromosomal regions on BMI are common in the Framingham study. When we accounted for these effects we were able to detect two new regions that showed significant linkage to BMI on chromosomes 10 and 16.

## Methods

### Design

The Framingham Heart Study (FHS) has been described in detail elsewhere [12]. Briefly, FHS divides the subjects into two recruitment groups. The first group is the original cohort of subjects, referred to as the 'cohort' here. This cohort consisted of the adult members between the ages of 28 and 62 in approximately 2/3 of the households in the town of Framingham, Massachusetts in 1948. Since then, the cohort has been examined every two years for 26 exams total. The second group primarily comprises the children of the original cohort and their spouses and is referred to as the 'offspring' here. The offspring were first examined in 1971–1974. They were next examined in 1979–1982 and every four years thereafter up to 1998, totaling six exams. In the early 1990s, DNA was extracted and shipped to the Mammalian Genotyping Service (MGS). The MGS produced genotypes for 1702 individuals in 330 families for 401 polymorphic markers (marker set 9, average heterozygosity 0.77) with an average intermarker spacing of 8.59 cM (SD = 3.97). The genotypes were checked for Mendelian consistency by the PEDSYS [13] program.

At each exam, each subject undergoes an extensive data gathering protocol. For this study we use only five variables: sex, age, height, weight, and cohort status. BMI was derived as weight (in kg) divided by the square of height (in meters).

Initially, we constructed six datasets corresponding to the six offspring exams matched to the cohort exam that corresponded in time [1]. These datasets varied in sample size due to individuals dropping out of the study. This dropout was primarily due to death. An objective of this study was to remove varying sample size as an explanation for the variation in lodscores across exams. Therefore *we constructed six new datasets that comprise only those individuals who had measurements on all six exams*. In each dataset, individuals who did not have phenotype measurements at all six exams were set to missing. Family structures were not changed. Logically, the individuals with data in these six new sets are the *intersection *of the individuals with phenotype data in the six original datasets. Therefore, we will refer to these six new datasets as the 'intersection datasets' and the six datasets that were used in our original linkage analysis [1] as the 'original datasets'. Note the datasets are numbered to indicate their temporal relationship; the individuals are youngest in dataset 1 and oldest in dataset 6. All subjects gave informed consent and the Institutional Review Board of the Boston University School of Medicine has approved all protocols.

### Statistical analysis

Linkage analysis was performed using a variance components approach [14,15] as implemented in Genehunter [16,17]. This approach uses the genotype information at a locus to decompose the phenotypic variance into a component attributable to the locus (known as a quantitative trait locus or QTL), a polygenic component and an environmental component. The genotype information at a locus is characterized by the probability that two related individuals share 0, 1, or 2 alleles identical by descent (IBD). All results are for multipoint linkage analysis. Genehunter will compute sex-specific means and can simultaneously incorporate the effects of covariates. For the genome scans on the full datasets we computed sex-specific means and included the effects of age, age^2^, and cohort status in the model.

Sex-specific linkage analysis for both sexes was performed by setting all phenotype values for one sex (or the other) to missing and performing the usual linkage analysis with the corresponding sex-specific mean.

All variance components are estimated by maximum likelihood. Linkage is tested by a likelihood ratio test in which a null hypothesis of the QTL variance component being equal to zero is compared to it being greater than zero. The resulting chi-square statistic is converted to a traditional lodscore by dividing by 2*ln(10). The proportion of the total phenotypic variation due to the QTL can be estimated, however, it has been shown [18,19] that the estimate of this effect is strongly correlated with the lodscore estimate and thus estimates the true effect poorly. Therefore, we will not present effect estimates here. Heritability estimates for all six of the BMI measures were obtained by variance components as implemented in SOLAR [15]. The Framingham Heart study is population-based and not selected for any particular trait; therefore no ascertainment correction is necessary.

### Randomization tests

There are significant differences in age between the original sets and the intersection sets (see Table 1). We performed randomization tests, at each point on the chromosome, to determine if the observed difference in maximum lodscore between the original and intersection datasets was significant. If significant, we have evidence, at this point on the chromosome, for a difference between the original sets and the intersection subsets. The inference that this difference is related to age is based on the observed age difference between the original sets and intersection subsets.

Let *e *be the number of individuals excluded from an original set to form an intersection set. First, we removed (set to missing) phenotype information on *e *individuals randomly selected (without regard to age) from the original set. Then we repeated the linkage analysis on this new set and retained the lodscores. We repeated this drop-then-link procedure for 10,000 replications, choosing a new set of size *e*, without replacement, randomly at each replication. The 10,000 linkage scans of this region form the null distribution of no effect of excluding *e *individuals from the original dataset. The p-value at each point in the region is the proportion of the 10,000 that are greater than or equal to the lodscore at that point in the intersection set.

We also performed randomization tests to determine if there were sex-specific effects of linked regions in the intersection sets. In each of the six intersection sets we constructed two subsets, one all men and one all women, and then performed the linkage on both subsets noting the difference in lodscores at each point. Under the null hypothesis of no sex-specific effect the difference in lodscores should be zero. If the difference in lodscores significantly deviates from the null hypothesis then we have evidence for a sex-specific effect at that point. Let *w *be the number of women in the full intersection set. First, we formed two subsets; in the first subset all phenotype information on *w *randomly selected individuals (from those individuals with complete data) was set to missing, while the second subset retained those same *w *individuals and set the other *n-w *individuals to missing. Then we repeated the linkage analysis on both of these new sets and retained the difference in lodscores at each point. We repeated this drop-then-link procedure on the two subsets for 10,000 replications, choosing a new set of size *w *randomly at each replication. The resulting 10,000 differences in lodscores at each point form the null distribution of no sex-specific effects of this region. The p-value at each point on the chromosome is the proportion of the 10,000 that are greater than or equal to the difference in the true sex-specific lodscores. These p-values will be plotted as -log_10_(p). While this p-value is for a one-tailed test, the chosen tail depends on which sex has the greater lodscore. The graph of this p-value will be labeled 'men' or 'women' to indicate which sex had the larger lodscore in the full intersection datasets. When both sexes had larger lodscore at different points in the region, we will plot the p-value both ways (see e.g. chromosome 7 in figure 5).

## Authors' contributions

LDA is the director of the Framingham Obesity Genetics project. He directed the analysis and wrote the manuscript. NLHC contributed to the design and performed most of the analysis. CSF is the physiologist on the Framingham Obesity Genetics project and provides expertise on the mechanism of obesity. CEJ contributed to the design, assisted in the analysis, and edited the manuscript. LAC is a senior statistical geneticist on the Framingham Heart Study and contributed to the design and edited the manuscript.
